# Retrograde installation of percutaneous transhepatic negative-pressure biliary drainage stabilizes pancreaticojejunostomy after pancreaticoduodenectomy: a retrospective cohort study

**DOI:** 10.1186/s12957-019-1645-1

**Published:** 2019-06-13

**Authors:** Chang Min Lee, Yong Joon Suh, Sam-Youl Yoon

**Affiliations:** 1 Department of Surgery, Korea University Medical Center Ansan Hospital, Ansan, 15355 Korea; 2 Department of Breast and Endocrine Surgery, Hallym University Sacred Heart Hospital, Anyang, 14068 Korea; 3 Department of Surgery, Hallym University Sacred Heart Hospital, 22, Gwanpyeong-ro 170 beon-gil, Dongan-gu, Anyang, 14068 Korea

**Keywords:** Pancreaticoduodenectomy, Pancreaticojejunostomy, Leakage, Fistula, Drainage

## Abstract

**Background:**

Leakage from the pancreatoenteric anastomosis has been one of the major complications of pancreaticoduodenectomy (PD). The aim of this study was to investigate the feasibility of retrograde installation of percutaneous transhepatic negative-pressure biliary drainage (RPTNBD), as part of which the drainage tube is intraoperatively inserted into the bile duct and afferent loop by surgical guidance to reduce pancreaticoenteric leakage after PD.

**Methods:**

We retrospectively reviewed the medical records of the patients who underwent pylorus-preserving PD or Whipple’s operation for a malignant disease between June 2012 and August 2016. We performed intraoperative RPTNBD to decompress the biliopancreatic limb in all patients and compared their clinical outcomes with those of internal controls.

**Results:**

Twenty-one patients were enrolled in this study. The operation time was 412.0 ± 92.8 min (range, 240–600 min). The duration of postoperative hospital stay was 39.4 ± 26.4 days (range, 13–105 days). Ten patients (47.6%) experienced morbidities of Clavien-Dindo grade > II, and 2 patients (9.5%) experienced pancreaticojejunostomy-related complications. The internal controls showed a higher incidence rate of pancreaticojejunostomy-related complications than the study participants (*P* = 0.020). Mortality occurred only in the internal controls.

**Conclusion:**

For stabilizing the pancreaticoenteric anastomosis after PD for a malignant disease, RPTNBD is a feasible and effective procedure. When PD is combined with technically demanding procedures, including hepatectomy or vascular reconstruction, RPTNBD could prevent fulminant anastomotic failure.

**Electronic supplementary material:**

The online version of this article (10.1186/s12957-019-1645-1) contains supplementary material, which is available to authorized users.

## Background

Pancreaticoduodenectomy (PD) is a standard procedure for treating malignancy in the pancreatic head and periampullary area. Because PD includes anastomoses, which requires an advanced surgical technique, the mortality rates associated with this procedure in the past few decades have been reported to be approximately 25–30% [1]. Although the mortality rate of patients undergoing PD has recently decreased owing to improvements in surgical techniques and perioperative management strategies, the postoperative morbidity rate is still as high as 40–50% [2]. One of the major complications of PD has been leakage from pancreaticoenteric anastomosis.

This morbidity frequently leads to a fatal course, because the leaked pancreatic juice may affect the surrounding structures. Digestive enzymes in the leaked fluid can also disrupt the other anastomoses (i.e., gastroenterostomy, enteroenterostomy, or choledochoenterostomy) and sometimes cause massive hemorrhage by eroding the vessels. These conditions delay the initiation of adjuvant chemotherapy, and eventually yielding poor long-term results, even though recent technologies provide effective interventions for controlling diverse complications. Thus, several procedures associated with PD have been investigated, with a focus on leakage prevention.

Strategies for preventing the incidence of pancreatic fistulas are divided into two categories. The first category includes procedures that reinforce the consistency of anastomosis. These are related to the methodology of anastomosis or pathway of pancreatic juice. There is no consensus regarding the clinical effectiveness of this category of strategies. For example, whether the dunking technique is superior or inferior to duct-to-mucosa anastomosis during pancreaticojejunostomy (PJ) cannot be concluded [3]. In addition, although recent studies showed the advantage of pancreaticogastrostomy (PG) over PJ in reducing the incidence of pancreatic fistula, no consensus has been reached regarding the issues of morbidity and mortality [4, 5].

Meanwhile, the second category includes several procedures that reduce the burden of pancreaticoenteric anastomosis. Some of these procedures are associated with the preoperative or intraoperative conditioning of the biliary tree, and others with stabilizing the anastomoses by special reconstruction methods. Still, others involve pancreatic or biliary decompression using the external drainage. In this regard, in June 2012, we introduced retrograde installation of percutaneous transhepatic negative-pressure biliary drainage (RPTNBD) for biliopancreatic decompression in patients who had undergone PD for malignant disease (i.e., duodenal cancer, pancreatic cancer, bile duct cancer, and other malignant conditions requiring PD for R0 resection). Preoperatively, endoscopic nasobiliary drainage (ENBD) was applied for biliary decompression. This novel procedure could minimize the leakage rates of pancreaticoenterostomy and decompress both PJ and choledochojejunostomy (CJ) simultaneously.

The aim of this study was to investigate the feasibility of RPTNBD by comparing clinical outcomes before and after the introduction of this technique.

## Methods

### Study design and participants

This was a retrospective cohort study performed in a single institute. We reviewed the medical charts of patients who underwent pylorus-preserving PD (PPPD) or Whipple’s operation for malignant disease between June 2012 and August 2016. Clinical outcomes of the patients were compared to those of internal controls. Internal controls included the patients who underwent PPPD or Whipple’s operation due to malignant disease before June 2012. Percutaneous transhepatic biliary drainage (PTBD) was not inserted in these patients postoperatively. PTBD insertion was not technically feasible because the biliary system was compressed in these patients. Only percutaneous abscess drainage (PAD) was inserted to remove the digestive juice that leaked from anastomoses. Approval to perform research on human subjects in this study was provided by the Institutional Review Board of Korea University Medical Center Ansan Hospital (registration number: 2018AS0029). This study adhered to the tenets of the Declaration of Helsinki.

### Procedures

The lymph nodes of the hepatoduodenal ligament, the celiac trunk, and the right side of the superior mesenteric artery were excised. We performed pancreaticoenteric resection, which included resection of the pancreatic head, duodenum, proximal jejunum (the first 15 cm from the ligament of Treitz), common bile duct, and gall bladder. The pancreas was divided with electrocautery, and the pancreatic duct was cut with Metzenbaum scissors. Bleeding of the cut surface was controlled by electrocauterization or suture ligation.

**Fig. 1:**
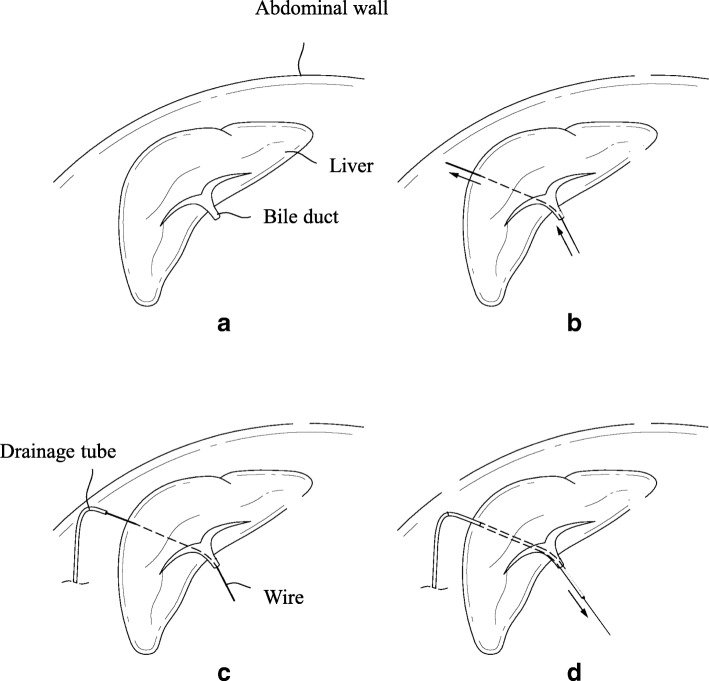
Surgical retrograde installation of percutaneous transhepatic negative-pressure biliary drainage. **a** The opening of the cut bile duct was identified for the insertion of a blunt-pointed probe. **b** The metal probe penetrated through the liver parenchyma. **c** A drainage tube was docked to the metal probe. **d** The drainage tube was retracted through the cut bile duct

**Fig. 2:**
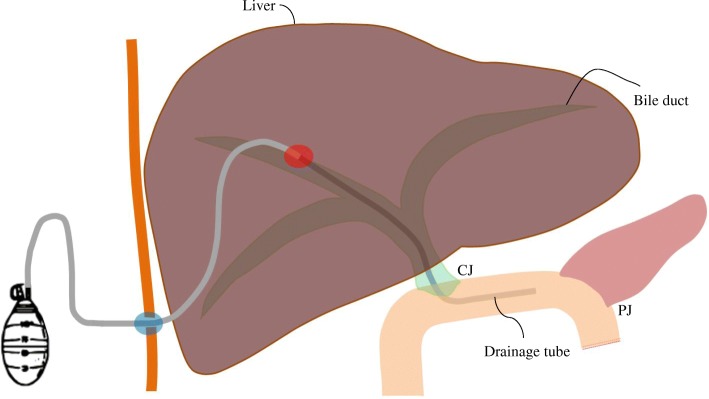
Final scheme of retrograde installation of percutaneous transhepatic negative-pressure biliary drainage. CJ choledochojejunostomy, PJ pancreaticojejunostomy

For pancreaticoenteric anastomosis, the divided jejunum was lifted through the mesocolon of the transverse colon (retrocolic approach). A duct-to-mucosa anastomosis was made between the pancreatic duct and the jejunal mucosa. A polyvinyl chloride (PVC) stent was inserted in the jejunal opening and pancreatic duct to stabilize the inner strength of the pancreaticoenteric anastomosis. Before starting CJ, we inserted a blunt-pointed probe into the cut bile duct. This probe was passed through the peripheral duct and pulled through the liver parenchyma. A PVC drain tube was docked to the blunt point of the probe and retracted through the cut bile duct (Fig. 1). An end-to-side anastomosis was made between the bile duct and the jejunum (distal from PJ). The retracted end of the PVC drain was inserted into the jejunum during CJ. The opposite end of the PVC drain was pierced through the abdominal wall and was connected to a low-vacuum silicone reservoir. The final scheme of RPTNBD is shown in Fig. 2. To restore the gastrointestinal continuity, Billroth II or Roux-en-Y reconstruction was performed. For Billroth II reconstruction, a Braun anastomosis was added.

### Assessments

Demographic, clinical, pathological, and therapeutic information was obtained from the medical records of the study participants. Outcomes, including the operation time, vascular reconstruction method, duration of postoperative hospital stay, time to the first semi-blend diet, and postoperative complications, were investigated. Postoperative complications were graded according to the Clavien-Dindo classification of surgical complications.

### Analysis

Internal controls were defined as patients, who underwent PPPD or Whipple’s operation for malignant disease without RPTNBD. Clinical, pathological, and therapeutic outcomes, including the incidence of PJ leakage, were compared between the study participants and the internal controls. In the present study, PJ leakage was defined as a drain output of any volume occurring on or after postoperative day 3 with an amylase level of at least three times the serum amylase levels [6].

### Statistical analyses

Patients with and without RPTNBD were compared using the chi-squared test or Fisher’s exact test for categorical data and the Student’s *t* test or Mann-Whitney *U* test for continuous data with abnormal distribution. In two-tailed tests, a *P* value < 0.05 was considered statistically significant. Statistical analysis was performed using SPSS 24.0 (SPSS Inc., Chicago, IL, USA).

## Results

Twenty-one patients underwent RPTNBD during PD for a malignant disease of the duodenum, common bile duct, or pancreas. The patients’ demographics are presented in Table 1. The mean age and BMI of the enrolled patients were 65.5 ± 11.2 years (range, 36–82 years) and 22.6 ± 4.1 kg/m^2^ (range, 15.3–33.3 kg/m^2^), respectively. Among the 21 enrolled patients, 13 (61.9%) underwent PPPD. The operation time was 412.0 ± 92.8 min (range, 240–600 min), and the duration of hospital stay was 39.4 ± 26.4 days (range, 13–105 days). The time to the first semi-blend diet was 8.4 ± 5.6 days (range, 3–31 days). The tumor size was 3.1 ± 1.2 cm (range, 1.1–5.4 cm). The numbers of retrieved and metastatic lymph nodes were 18.0 ± 8.1 (range, 2–36) and 1.6 ± 2.8 (range, 0–8), respectively. Thirteen patients showed postoperative morbidities. Among the 13 cases, 10 (47.6%) corresponded to a morbidity of Clavien-Dindo grade III or higher (Table 2). Four patients underwent radiological interventions for fluid collection around the PJ or CJ sites (cases 7, 11, 14, and 16); however, tubographic images acquired via the RPTNBD pathway showed no association between PJ and the fluid collection. All the patients recovered with conservative treatment.

**Table 1:** Demographic data of the RPTNBD group in the present study

Number	Age	Sex	BMI	ASA score	Preoperative ENBD	Diagnosis
1	78	Male	23.4	III	No	Pancreatic head cancer
2	56	Male	21.3	II	No	Pancreatic head cancer
3	82	Male	24.7	III	No	Pancreatic head cancer
4	66	Male	23.3	II	Yes	CBD cancer
5	36	Male	17.9	II	No	Pancreatic head cancer
6	77	Female	20.7	II	Yes	CBD cancer
7	53	Male	21.2	II	No	Klatskin tumor
8	75	Male	19.7	III	Yes	Pancreatic head cancer
9	60	Male	18.4	III	Yes	Pancreatic head cancer
10	78	Female	27.8	II	No	Pancreatic head cancer
11	68	Male	23.4	II	No	Pancreatic head cancer
12	72	Female	15.3	II	Yes	Pancreatic head cancer
13	60	Male	20.7	II	No	Pancreatic head cancer
14	71	Male	27.0	II	Yes	AOV cancer
15	72	Male	24.3	II	No	CBD cancer
16	57	Female	33.3	III	No	Klatskin tumor
17	68	Male	25.0	III	No	CBD cancer
18	76	Female	26.2	II	No	AOV cancer
19	58	Male	19.3	II	Yes	Pancreatic head cancer
20	54	Female	17.7	III	No	Pancreatic head cancer
21	59	Male	24.2	II	No	CBD cancer

*BMI* body mass index, *ASA* American Society of Anesthesiologists, *ENBD* endoscopic nasobiliary drainage, *CBD* common bile duct, *AOV* ampulla of Vater

**Table 2:** Clinicopathologic data of the RPTNBD group in the current study

Number	Operation	Vascular reconstruction	Operation time (min)	Hospital stay (day)	Time to SBD (day)	C-D classification	Pathology	RLN	MLN
1	PPPD	.	350	16	8	0	NEC	2	0
2	PPPD	.	517	27	6	II	AC	14	5
3	Whipple	.	440	15	6	0	AC	21	0
4	PPPD	.	340	31	6	II	AC	22	0
5	PPPD	.	517	13	6	0	AC	16	0
6	PPPD	.	380	18	5	0	AC	20	0
7	HPD^a^	.	450	30	12	IIIa	XGC	15	0
8	PPPD	PV, RHA	360	22	8	0	AC	19	8
9	PPPD	PV, RHA	360	36	7	IIIa	ACC	10	0
10	PPPD	.	350	52	10	IIIa	AC	26	8
11	PPPD	PV	498	104	31	IIIa	ACC	16	0
12	Whipple	.	327	23	5	0	AC	20	0
13	PPPD	.	360	20	7	0	AC	4	1
14	PPPD	.	330	48	7	IIIa	AC	15	0
15	Whipple	PV	470	105	10	IIIa	AC	36	6
16	HPD^b^	.	600	56	11	IIIa	AC	25	1
17	PPPD	.	570	26	7	IIIa	AC	10	0
18	PPPD	.	240	65	8	IIIa	AC	20	0
19	Whipple	PV, CHA	440	46	7	II	AC	13	1
20	Whipple	PV	440	52	3	IIIa	AC	21	4
21	Whipple	.	314	22	7	0	AC	32	0

*SBD* semi-blend diet, *C-D* Clavien-Dindo, *RLN* retrieved lymph nodes, *MLN* metastatic lymph nodes, *PPPD* pylorus-preserving pancreatoduodenectomy, *NEC* neuroendocrine carcinoma, *AC* adenocarcinoma, *HPD* hepatopancreatoduodenectomy, *XGC* xanthogranulomatous cholecystitis, *PV* portal vein, *RHA* right hepatic artery, *ACC* acinar cell carcinoma, *CHA* common hepatic artery

^a^This patient underwent Whipple’s operation and right hemi-hepatectomy

^b^This patient underwent Whipple’s operation, right hemi-hepatectomy, and S1 segmentectomy

The demographics of the internal control are presented in Table 3. The mean age and BMI of the enrolled patients were 62.6 ± 11.4 years (range, 30–78 years) and 22.6 ± 3.5 kg/m^2^ (range, 17.2–32.4 kg/m^2^), respectively. Among the 31 patients in the internal control group, 20 (64.5%) underwent PPPD. The operation time was 420.2 ± 170.4 min (range, 267–1,015 min) and the duration of hospital stay was 30.3 ± 22.5 days (range, 9–118 days). The time to the first semi-blend diet was 12.0 ± 12.4 days (range, 4–61 days). The tumor size was 3.2 ± 1.4 cm (range, 0.8–5.8 cm). The numbers of retrieved and metastatic lymph nodes were 19.7 ± 6.7 (range, 4–36) and 1.5 ± 2.4 (range, 0–8), respectively. Twenty-two patients had postoperative morbidities. Among the 22 cases, 10 (52.4%) corresponded to a morbidity of Clavien-Dindo grade III or higher (Table 4). As shown in Table 5, the incidence of postoperative complications did not differ between the study participants and the internal controls (*P* = 0.494). However, the internal controls showed a higher incidence of PJ complications than the study participants (*P* = 0.020). Mortality occurred in the internal controls, although 12 (38.7%) patients with PJ complication underwent radiological interventions of PAD to remove the digestive juice leaked from the anastomoses. The internal control group showed higher morbidity and mortality rates than the RPTNBD group (Additional file 1).

**Table 3:** Demographic data of the internal controls in the present study

Number	Age	Sex	BMI	ASA score	Preoperative ENBD	Diagnosis
1	Male	63	23.3	II	No	Pancreatic head cancer
2	Male	52	18.8	II	No	CBD cancer
3	Female	59	32.4	II	Yes	Pancreatic head cancer
4	Female	57	24.1	II	No	CBD cancer
5	Male	51	21.0	II	No	Pancreatic head cancer
6	Male	52	24.0	II	Yes	Pancreatic head cancer
7	Male	45	23.6	I	No	Duodenal cancer
8	Male	50	18.8	II	Yes	Duodenal cancer
9	Male	76	24.1	II	No	CBD cancer
10	Male	69	21.9	II	No	CBD cancer
11	Female	76	18.1	II	No	CBD cancer
12	Female	73	22.9	III	Yes	Pancreatic head cancer
13	Female	57	23.4	I	No	Pancreatic head cancer
14	Male	75	21.3	II	Yes	AOV cancer
15	Female	73	29.1	II	Yes	CBD cancer
16	Male	53	26.6	II	No	Pancreatic head IPMN
17	Female	30	17.2	I	No	Pancreatic head cancer
18	Male	74	25.7	II	No	Pancreatic head cancer
19	Male	62	24.1	III	Yes	Pancreatic head IPMN
20	Female	72	23.1	III	No	Pancreatic head cancer
21	Female	58	23.8	II	No	CBD cancer
22	Male	77	23.1	II	No	CBD cancer
23	Male	71	18.5	II	Yes	Pancreatic head cancer
24	Male	58	24.8	II	No	Pancreatic head cancer
25	Male	66	18.7	II	Yes	Pancreatic head cancer
26	Male	50	18.2	II	No	AGC
27	Female	60	20.0	II	No	Duodenal GIST
28	Male	59	18.8	II	No	Pancreatic head cancer
29	Male	65	25.3	III	No	AOV cancer
30	Male	72	27.4	II	Yes	CBD cancer
31	Female	78	19.8	II	Yes	CBD cancer

*BMI* body mass index, *ASA* American Society of Anesthesiologists, *ENBD* endoscopic nasobiliary drainage, *CBD* common bile duct, *AOV* ampulla of Vater, *IPMN* intraductal papillary mucinous neoplasm, *AGC* advanced gastric cancer, *GIST* gastrointestinal stromal tumor

**Table 4:** Clinicopathologic data of the patients in the internal control

Number	Operation	Vascular reconstruction	Operation time (min)	Hospital stay (day)	Time to SBD (day)	C-D classification	Pathology	RLN	MLN
1	Whipple	.	345	42	6	IIIa	AC	22	0
2	PPPD	.	275	15	6	0	AC	14	1
3	PPPD	.	267	14	9	0	AC	19	0
4	PPPD	.	330	70	39	IIIb	AC	26	0
5	PPPD	.	400	14	6	0	NEC	16	0
6	PPPD	.	480	66	8	IIIa	AC	22	4
7	Whipple	.	365	12	7	0	AC	13	0
8	Whipple	.	765	31	6	IIIa	AC	19	8
9	PPPD	.	370	9	7	0	AC	11	0
10	PPPD	.	330	15	7	0	AC	24	8
11	PPPD	.	370	17	12	II	AC	10	0
12	PPPD	.	350	15	7	0	AC	20	0
13	PPPD	.	340	44	33	IVa	NEC	4	1
14	PPPD	.	505	41	7	IIIa	AC	18	0
15	PPPD	.	310	24	22	II	AC	36	3
16	PPPD	.	1015	30	.	V	AC	25	1
17	PPPD	.	570	118	61	IIIb	AC	32	0
18	PPPD	.	490	23	13	II	AC	20	0
19	PPPD	.	290	23	15	II	AC	13	0
20	Whipple	.	355	41	4	II	AC	21	4
21	PPPD	.	327	31	6	IIIa	AC	15	0
22	Whipple	.	362	53	8	V	AC	17	0
23	Whipple	PV	467	31	9	V	AC	21	6
24	PPPD	.	313	23	8	IIIa	AC	24	0
25	Whipple	PV	755	10	.	V	AC	28	4
26	Whipple	.	650	34	9	II	AC	18	0
27	Whipple	.	285	19	5	II	AC	13	0
28	Whipple	.	365	19	6	0	NEC	21	0
29	Whipple	.	365	23	7	IIIa	AC	19	4
30	PPPD	.	318	19	7	0	AC	21	1
31	PPPD	.	297	14	7	II	AC	29	0

*SBD* semi-blend diet, *C-D* Clavien-Dindo, *RLN* retrieved lymph nodes, *MLN* metastatic lymph nodes, *PPPD* pylorus-preserving pancreatoduodenectomy, *NEC* neuroendocrine carcinoma, *AC* adenocarcinoma, *PV* portal vein

**Table 5:** Comparison of outcomes between the RPTNBD group and internal control group

	RPTNBD group (*n* = 21)	Internal control group (*n* = 31)	*P*
Age (years), means ± SD	65.5 ± 11.2	62.6 ± 11.4	0.330
Female (%)	28.6	35.5	0.765
BMI (kg/m^2^), means ± SD	22.6 ± 4.1	22.6 ± 3.5	0.980
PPPD (%)	61.9	64.5	1.000
Preoperative ENBD (%)	33.3	35.5	1.000
Operation time (min), means ± SD	412.0 ± 92.8	420.2 ± 170.4	0.843
Hospital stay (days), means ± SD	39.4 ± 26.4	30.3 ± 22.5	0.190
Time to SBD (day), means ± SD	8.4 ± 5.6	11.3 ± 11.7	0.307
Vascular reconstruction (%)	33.3	6.5	0.012
Hepatectomy (%)	9.5	3.2	0.339
Postoperative PAD (%)	19.0	45.2	0.076
Fluid collection (%)	19.0	6.5	0.207
Anastomotic leakage (%)	0	38.7	0.001
Morbidity (%)	61.9	71.0	0.494
C-D grade > II (%)	47.6	45.2	1.000
PJ complication (%)	9.5^a^	38.7	0.020
Mortality (%)	0.0	12.9	0.087

*SD* standard deviation*, BMI* body mass index*, PPPD* pylorus-preserving pancreatoduodenectomy, *ENBD* endoscopic nasobiliary drainage*, SBD* semi-blend diet, *PAD* percutaneous abscess drainage, *C-D* Clavien-Dindo, *PJ* pancreaticojejunostomy

^a^These patients had only fluid collection around PJ sites with no evidence of leakage in tubography

## Discussion

Considering the results, we believe that RPTNBD might contribute to the salvage treatment of a morbidity after PD. Because the present study included far advanced cases that required some challenging procedures (i.e., major vessel reconstruction or simultaneous hepatectomy) to accomplish R0 resection, several cases carried a high risk of morbidity or mortality. However, most postoperative complications were managed with intravenous antibiotics and additional PAD. One patient who underwent portal vein and right hepatic artery reconstructions did not show any postoperative morbidity. It was remarkable that no mortality occurred even in the advanced cases that required technically demanding procedures.

Because the corrosive property of pancreatic juice might cause secondary catastrophes in the surgical field, several strategies have been designed to prevent pancreaticoenteric leakage after the introduction of PD. Although many strategies have been established for the postoperative safety of PD, biliary tract decompression is one of the most traditional methods that reduces the morbidity rate of PD. In 1935, Whipple et al. first proposed preoperative biliary drainage (PBD), by which obstructive jaundice could be corrected in patients with periampullary lesions [7]. Preoperative correction of jaundice could be related to the clinical outcomes of patients undergoing PD, because hyperbilirubinemia is associated with impaired liver function, coagulation disorder, compromised immunity, accumulation of circulating endotoxin, and wound problems [8–11]. Currently, PBD has been facilitated by the technical advancement of radiological interventions (i.e., PTBD) and endoscopic procedures. With regard to the clinical outcomes of patients undergoing PD, some studies showed the benefits of PBD, including improved resection rate, morbidity, and mortality rates [12, 13]. However, other reports indicated drawbacks of this procedure. Several researchers reported the possibility of hyperamylasemia after radiological or endoscopic procedures [14, 15]. In addition, some comparative studies revealed that PBD caused certain morbidities rather than advantages in patients who underwent PD [16, 17]. Therefore, the benefit of performing PBD before PD is not yet established.

Biliary drainage can be performed intraoperatively. Doi et al. reported an intraoperative biliary decompression technique in which a newly developed curved drainage clamp (Mizuho Co., Tokyo, Japan) was used for the drainage of the common hepatic duct stump [18]. However, it was difficult to determine the effect of this technique on anastomosis, despite the possibility that this technique reduces the risk of hepatic complications. As part of another biliary decompression technique, the special structures are added after PD. Two strategies were used for adding these special structures over the last few decades. Braun anastomosis is one of these two strategies; it reduces the pressure in the biliopancreatic limb to avoid the afferent loop syndrome. The result of a randomized clinical trial showed that Braun anastomosis might decrease the pressure in the biliopancreatic limb after standard Whipple’s operation [19]. Separating anastomoses is the other strategy. Isolated Roux loop PJ was performed to lower the incidence rate of pancreatic fistula [20]. Double Roux-en-Y reconstruction was proposed to isolate pancreaticoenteric, choledochoenteric, or gastroenteric anastomosis [21]. However, all these modified structures rendered no significant protection against pancreaticoenteric leakage [19–21].

When PJ failures are diagnosed postoperatively, several radiological interventions can be helpful in maintaining the conservative treatment. PTBD and PAD are the representative procedures that have been widely accepted in the clinical field. These interventions can minimize anastomotic soling. PTBD reduces biliary flow into the afferent loop, which effectively decreases pressure in the disrupted anastomosis. PAD, on the contrary, may remove the digestive juice that has already leaked from the PJ or CJ site. Currently, the conservative strategy for the management of PJ or CJ failure is usually composed of PTBD or PAD, when radiological interventions can be performed under the guidance of real-time imaging techniques. Although PTBD heals the failed anastomosis by reducing the leakage, this intervention depends on biliary imaging. If the biliary ducts are not dilated, postoperative PTBD is technically demanding. Therefore, we performed RPTNBD intraoperatively. Our novel method was designed by incorporating the advantages of the previous procedures. The biliary decompression effect of RPTNBD may be equal to that of PTBD; however, the former does not require radiological guidance. As RPTNBD is intraoperatively performed during PD, surgeons can insert the drainage tube into the biliary duct.

RPTNBD has a protective effect against anastomotic leakage in both PJ and CJ sites. Similar to PTBD, RPTNBD decreases the high pressure of the afferent loop resulting from the accumulation of bile or pancreatic juice, which inevitably occurs during the paralytic ileus period after PD. When a minor leakage occurs in the PJ or CJ site, RPTNBD can reduce the risk of anastomotic failure. Although PAD had to be applied for fluid collection around the PJ or CJ site in several cases (cases 7, 11, 14, and 16) in the present study, these morbidities did not lead to the fulminant failure of PJ or CJ. Their drain amylase levels did not exceed three times the serum amylase levels. Tubography is also possible via the RPTNBD route, which can facilitate making a critical decision in the postoperative course (Additional file 2). For example, although computed tomography implied complicated fluid collection around the pancreas in two cases (cases 7 and 11), in our study, we could confirm no connection between the fluid collection and PJ using tubography via RPTNBD. In such cases, tubography via RPTNBD could provide an important clue to avoid unnecessary delay of the clinical decision.

One limitation of RPTNBD is that the surgeon should have reliable knowledge regarding the hepato-biliary anatomy. This is also an important precondition for performing RPTNBD. Although we did not encounter any accidental hemorrhage, introducing a probe into the intrahepatic bile duct may harbor a risk of injury to the hepatic structures.

## Conclusion

In conclusion, if a skilled surgeon performs RPTNBD, pancreaticoenteric anastomosis may be stabilized after PD. RPTNBD is expected to be effective in minimizing PJ or CJ anastomotic failure, which can arise in compromised patients.

## Additional files

Additional file 1: **Table S1.** Comparison of outcomes between RPTNBD group and internal controls with PAD related to anastomotic leakage. *SD* standard deviation*, BMI* body mass index*, PPPD* pylorus-preserving pancreatoduodenectomy, *ENBD* endoscopic nasobiliary drainage*, SBD* semi-blend diet, *PAD* percutaneous abscess drainage, *C-D* Clavien-Dindo, *PJ* pancreaticojejunostomy. (DOCX 26 kb)

Additional file 2: **Figure S1.** Tubography showing each anastomosis via the route of retrograde installation of percutaneous transhepatic negative-pressure biliary drainage. *CJ* choledochojejunostomy, *PJ* pancreaticojejunostomy, *GJ* gastrojejunostomy. (PPTX 1740 kb)

## Data Availability

The authors presented all the necessary information about the study in the manuscript and its supplementary material.

## Abbreviations

BMI: Body mass index
CJ: Choledochojejunostomy
PAD: Percutaneous abscess drainage
PBD: Preoperative biliary drainage
PD: Pancreaticoduodenectomy
PG: Pancreaticogastrostomy
PJ: Pancreaticojejunostomy
PPPD: Pylorus-preserving pancreaticoduodenectomy
PTBD: Percutaneous transhepatic biliary drainage
PVC: Polyvinyl chloride
RPTNBD: Retrograde installation of percutaneous transhepatic negative-pressure biliary drainage
