# Supplementary material for: Retrograde installation of percutaneous transhepatic negative-pressure biliary drainage stabilizes pancreaticojejunostomy after pancreaticoduodenectomy: a retrospective cohort study
Source: World J Surg Oncol. 2019 Jun 13;17:101. doi: 10.1186/s12957-019-1645-1 (PMC6567420; doi:10.1186/s12957-019-1645-1)
Supplement: Supplementary file 1 — Table S1. Comparison of outcomes between RPTNBD group and internal controls with PAD related to anastomotic leakage. SD standard deviation, BMI body mass index, PPPD pylorus-preserving pancreatoduodenectomy, ENBD endoscopic nasobiliary drainage, SBD semi-blend diet, PAD percutaneous abscess drainage, C-D Clavien-Dindo, PJ pancreaticojejunostomy. (DOCX 26 kb) [file 12957_2019_1645_MOESM1_ESM.docx]

**Table S1** Comparison of outcomes between RPTNBD group and internal controls with PAD related to anastomotic leakage

|  | RPTNBD group (n = 21) | Internal controls (n = 12) | *P* |
| --- | --- | --- | --- |
| Age (years), means ± SD | 65.5 ± 11.2 | 59.3 ± 12.9 | 1.000 |
| Female (%) | 28.6 | 25.0 | 1.000 |
| BMI (kg/m^2^), means ± SD | 22.6 ± 4.1 | 22.1 ± 3.15 | 0.980 |
| PPPD (%) | 61.9 | 58.3 | 1.000 |
| Preoperative ENBD (%) | 33.3 | 41.7 | 0.716 |
| Operation time (minutes), means ± SD | 412.0 ± 92.8 | 521.2 ± 220.0 | 0.843 |
| Hospital stay (days), means ± SD | 39.4 ± 26.4 | 43.9 ± 29.3 | 0.190 |
| Time to SBD (day), means ± SD | 8.4 ± 5.6 | 15.9 ± 18.7 | 0.307 |
| Vascular reconstruction (%) | 33.3 | 16.7 | 0.429 |
| Hepatectomy (%) | 9.5 | 0 | 0.523 |
| Postoperative PAD (%) | 19.0 | 100 | < 0.001 |
| Fluid collection (%) | 19.0 | 0 | 0.271 |
| Anastomotic leakage (%) | 0 | 100 | < 0.001 |
| Morbidity (%) | 61.9 | 100 | 0.030 |
| C-D grade > II (%) | 47.6 | 100 | 0.002 |
| PJ complication (%) | 9.5^a^ | 100 | < 0.001 |
| Mortality (%) | 0.0 | 33.3 | 0.012 |

^a^These patients had only fluid collection around PJ sites with no evidence of leakage in tubography.

*SD* standard deviation*, BMI* body mass index*, PPPD* pylorus-preserving pancreatoduodenectomy, *ENBD* endoscopic nasobiliary drainage*, SBD* semi-blend diet, *PAD* percutaneous abscess drainage, *C-D* Clavien-Dindo, *PJ* pancreaticojejunostomy
