# Supplementary figures and images for: Retrograde installation of percutaneous transhepatic negative-pressure biliary drainage stabilizes pancreaticojejunostomy after pancreaticoduodenectomy: a retrospective cohort study
Source: World J Surg Oncol. 2019 Jun 13;17:101. doi: 10.1186/s12957-019-1645-1 (PMC6567420; doi:10.1186/s12957-019-1645-1)

## Slide 1
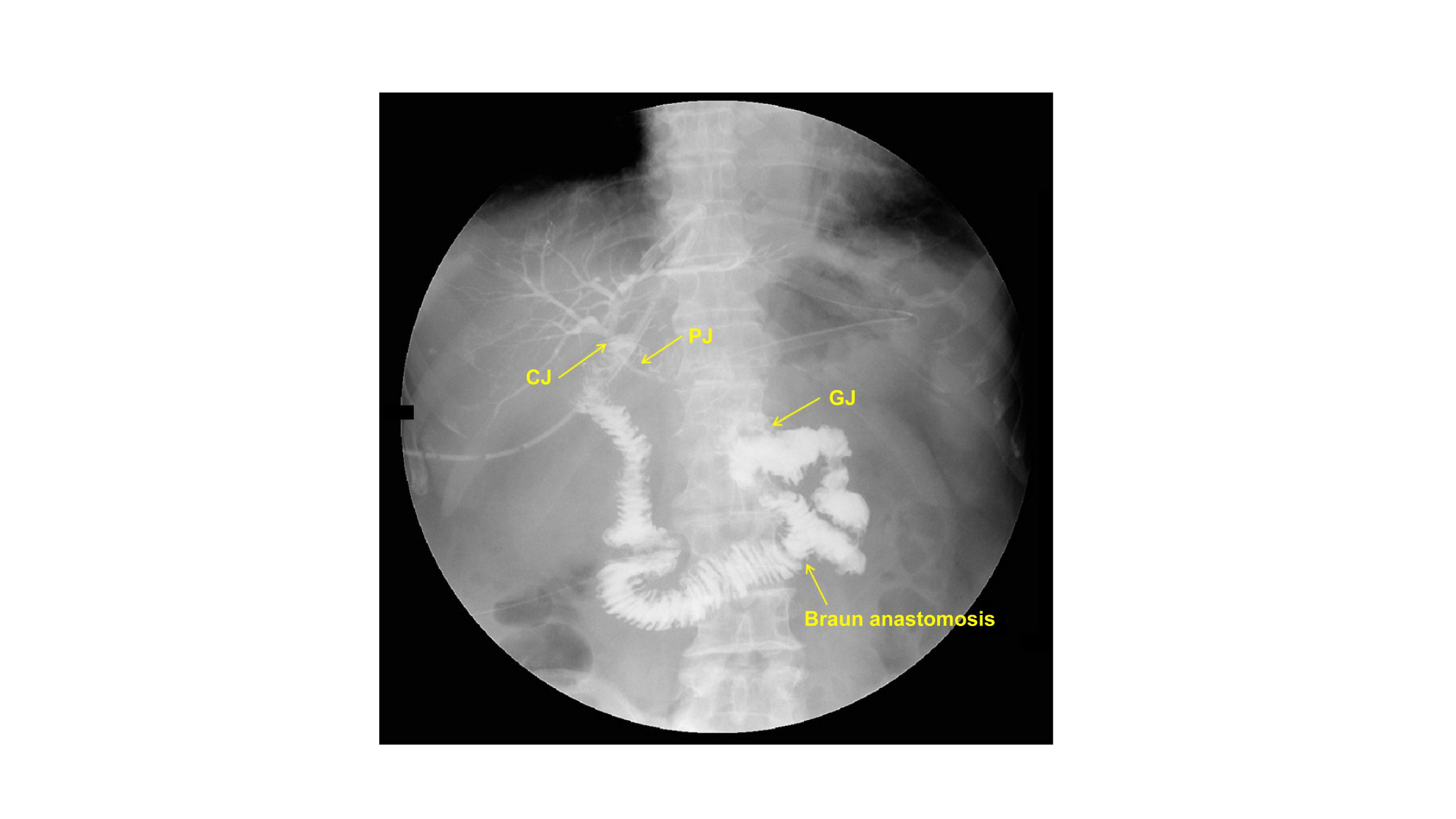

Supplement: Supplementary file 2 — Figure S1. Tubography showing each anastomosis via the route of retrograde installation of percutaneous transhepatic negative-pressure biliary drainage. CJ choledochojejunostomy, PJ pancreaticojejunostomy, GJ gastrojejunostomy. (PPTX 1740 kb) [file 12957_2019_1645_MOESM2_ESM.pptx]
